# Role of *Tsukamurella* species in human infections: first literature review

**DOI:** 10.1016/j.nmni.2017.10.002

**Published:** 2017-10-10

**Authors:** S. Safaei, M. Fatahi-Bafghi, Omid Pouresmaeil

**Affiliations:** 1) International Campus, Iran; 2) Department of Microbiology, Faculty of Medicine, Shahid Sadoughi University of Medical Sciences, Yazd, Iran

**Keywords:** Isolation, molecular identification, taxonomy, *Tsukamurella*

## Abstract

*Tsukamurella* is an aerobic, Gram-positive and nonmotile bacterium. It was first isolated in 1941 from the mycetoma and ovaries of the bedbug. The primary strains were named *Corynebacterium paurometabolum* and *Gordona aurantiaca* and are different from the Collins *et al.*, 1988 classification of the new *Tsukamurella* genus. Human infections with *Tsukamurella* species are rare because the species is a kind of saprophyte bacterium; however, most information regarding this species comes from case reports. Molecular markers for the identification *Tsukamurella* include sequencing of 16S rRNA, *groEL, rpoB, secA1* and *ssrA* genes. Given the lack of information on the treatment of *Tsukamurella* infections, a combination of various antibiotic agents is recommended.

## Introduction

Actinomycetes that have mycolic acid (chemotype IV) have been classified under genera such as *Corynebacterium, Gordonia, Mycobacterium, Nocardia, Rhodococcus, Tsukamurella, Skermania* and *Williamsia* [1]. As determined by 16S rRNA gene sequencing, these taxa have numerous features; *Corynebacterineae* comprise all these taxa, along with *Turicella* [2]. The genus *Tsukamurella* is an aerobic actinomycetes with a series of very long chains and unsaturated mycolic acid. It belongs to *Actinobacteria, Actinomycetales, Corynebacterineae* and *Tsukamurellaceae. Tsukamurella* was introduced in 1988 by Collins *et al.* according to the analysis of Japanese scientist Michico Tsukamura and of the studies of Steinhaus [3], [4], [5], [6]. *Tsukamurella paurometabola* was the first member of this genus, and it has a complex history. It was transferred from the genus *Gordona* to *Rhodococcus* and eventually classified as the genus *Tsukamurella.* The name *Tsukamurella* was selected to honor Michio Tsukamura, and *paurometabolum* (*pauros,* ‘small,’ and *metabolos,* ‘changeable’) was chosen because it had been described previously by Steinhaus [5] and refers to the fact that this bacterium is inactive in most phenotypic tests. On the basis of the rules of nomenclature, the name was then changed to *paurometabola* [4], [7]. The genus *Tsukamurella* consists of 11 species with available published names (http://www.bacterio.net/tsukamurella.html) [9]. *Tsukamurella* spp. are environmental saprophytes which are isolated from soil, arthropods, water, sludge foam and sponges [9]. They are opportunistic pathogens and can be spread through clinical instruments (e.g. catheters). They therefore can cause various infections in humans, such as pulmonary and cutaneous infections and meningitis; colonization also occurs in immunosuppressed individuals [1]. To date, nine species of the genus *Tsukamurella* have been isolated from human infections: *inchonensis, paurometabola, strandjordii, tyrosinosolvens, pulmonis, hongkongensis* and *sinensis* [10], [11]. Although *Tsukamurella serpentis* was recently isolated from the oral cavity of two venomous snakes (*Naja atra*) in China, there is no report of human infection by this species after being bitten [12].

## History of *Tsukamurella*

One member of the aerobic actinomycetes with a series of very long chains and unsaturated mycolic acids that was created in 1988 is *Tsukamurella. Tsukamurella* is a Gram-positive, rod-shaped bacterium that is typically misidentified as *Corynebacterium, Rhodococcus, Nocardia* and some nontuberculous mycobacteria species, so molecular methods are necessary for their accurate identification. Members of the taxa that comprise mycolic acids with wall chemotype IV differ in some features; *Tsukamurella* consists of 64 to 78 carbon atoms, *Nocardia* 44 to 60, *Mycobacterium* 60 to 90 and *Rhodococcus* 34 to 64. The G+C content of *Nocardia* is 64 to 72 mol%, *Mycobacterium* 62 to 70 mol% and *Rhodococcus* 63 to 73 mol%, but *Tsukamurella* is 67 to 68 mol%, although some species are exceptions to these ranges. Tuberculostearic acid is another attribute that may be used to differentiate among genera. *Tsukamurella, Nocardia, Mycobacterium* and *Rhodococcus* include tuberculostearic acid, but *Corynebacterium* does not—again, with the exception of some species [4]. Steinhaus [5] in 1941 isolated an organism from the mycetoma and ovaries of bedbugs (*Cimex lectularius*) and named it *Corynebacterium paurometabolum.* The presence of *meso*-diaminopimelic acid and an arabinogalactan polymer also causes misidentification because strains that contain these substances resemble *Corynebacterium.* Unsaturated mycolic acid (68 to 76 carbon atoms and two to six double bonds) can be used to distinguish *Tsukamurella* species from *Corynebacterium* [4]. Tsukamura and Mizuno [6] in 1971 identified a similar species with long mycolic acid as *Gordona aurantiaca. Rhodococciis aurantiacus* was initially classified under the genus *Rhodococcus,* but because it did not have features that can be distinctly associated with the genus *Rhodococcus,* the species was omitted from this classification. The findings of Goodfellow *et al.* showed that *Tsukamurella* is similar to *Mycobacterium* and *Nocardia,* but because of very long series and unsaturated mycolic acid, it can differ from other mycolic acid–containing actinomycetes [4].

In accordance with the results of 16S rRNA gene analyses, Collins *et al.* [4] in 1988 reclassified *C. paurometabolum* and *R. aurantiacus* under a new genus, *Tsukamurella.* Apart from being a Gram-positive bacterium*, Tsukamurella* is a partially acid fast, obligatory aerobic bacterium that does not have a capsule or aerial hyphae. It is chemoorganotrophic, catalase positive and pyrazinamidase positive, and some species can produce acid of some sugars. Moreover, it is a non–spore forming, lysozyme-resistant bacterium with nonmotile, rod-shaped cells that can be straight or curved and can be seen in pairs or groups of coccobacillary forms by Gram stain. Some *Tsukamurella* species produce pigmented colonies; isolation by Löwenstein-Jensen and brain–heart infusion media indicated that *Tsukamurella* colonies are different. The species are long rods at the first stage of growth but later separate and form new rods. Colonies are visible at 24 to 72 hours' growth [13]. The optimal growth temperature of *Tsukamurella* is 25 to 35°C, although there are some exceptions (Table 1) [3]. The cell envelope of the bacterium consists of peptidoglycan, sugars (arabinose and galactose, as shown in a sugar analysis), phospholipids, fatty acids, mycolic acid and unsaturated menaquinones. Cell wall analysis has shown *meso*-diaminopimelic acid type A1 in the structure of peptidoglycan. The species also contains phosphatidyl ethanolamine and 10-methyloctadecanoid fatty acids, and it has a G+C content of 67 to 68 mol% [14]. Peptidoglycan consists of d-alanine, l-alanine, *N*-acetylglucosamine, d-glutamic acid and muramic acid. Phospholipids contain phosphatidylinositol, phosphatidylethanolamine and diphosphatidylglycerol. Fatty acids contain tuberculostearic acids [15].

**Table 1:** Some phenotypic characteristics of *Tsukamurella* spp

Name	OT (°C)	NaCl concentration (%)	Enzyme				Carbon utilization											Hydrolysis								Study
			Oxi	Cata	Nit	Lip	Arab	Fru	Man	Sorb	Xly	Gala	Cello	Arabi	Dul	*meso*-Ery	Sali	Xanthine	Adenine	Hypoxanthine	Casein	Aesculin	Urea	Tyrosine	Tween 80	
*T. hongkongensis*	ND	ND	−	+	ND	ND	−	ND	+	+	−	+	ND	+	ND	ND	ND	ND	ND	ND	ND	ND	ND	ND	ND	[60]
*T. inchonensis*	24–45	5%	ND	ND	−	ND	+	−	+	+	+	−	−	−	−	−	+	−	ND	+	−	+	+	−	ND	[61]
*T. paurometabola*	10–35	ND	ND	ND	ND	ND	−	+	−	+	+	−	ND	−	−	−	+	−	−	ND	−	ND	+	−	+	[4]
*T. pseudospumae*	25–30	ND	+	ND	ND	ND	+	+	−	+	+	−	−	+	−	−	−	−	ND	−	ND	+	−	+	ND	[62]
*T. pulmonis*	24–37	ND	ND	+	−	ND	ND	ND	+	+	−	ND	+	+	+	+	+	−	ND	+	−	−	+	−	+	[52]
*T. sinensis*	ND	ND	−	+	ND	ND	−	ND	−	−	−	−	ND	−	ND	ND	ND	ND	ND	ND	ND	ND	ND	ND	ND	[60]
*T. soli*	30	3.0%	−	+	−	+	−	+	ND	+	ND	ND	−	+	−	−	+	−	ND	−	−	+	+	+	+	[63]
*T. spumae*	25–37	ND	+	ND	ND	ND	+	−	+	+	+	−	+	+	+	+	−	−	ND	+	ND	ND	+	+	ND	[64]
*T. strandjordae*	28–35	5%	ND	+	ND	ND	−	+	+	+	ND	+	ND	+	ND	ND	ND	ND	ND	ND	ND	ND	ND	ND	ND	[11]
*T. tyrosinosolvens*	24–37	5%	ND	ND	ND	ND	−	ND	+	+	−	+	−	ND	ND	−	ND	+	−	+	−	+	+	+	+	[65]
*T. serpentis*	10,37	7%	−	+	ND	ND	+	+	+	+	+	+	ND	+	+	+/−	ND	−	ND	ND	ND	+	ND	−	ND	[12]

+/− indicates different utilization.

Arab, arabinose; Arabit, arabitol; Cata, catalase; Cello, cellobiose; Dul, dulcitol; Fru, fructose; Gala, galactose; Lip, lipase; Man, mannitol; *meso*-Ery, *meso*-erythritol; ND, not determined; Nit, nitrate reductase; OT, optimum temperature; Oxi, oxidase; Sali, salicin; Sorb, sorbitol; Xly, xylose.

## Infections Caused by *Tsukamurella* Species

To date there has been no decisive report that shows any specific virulence factor for this genus. Most information regarding human infection by *Tsukamurella* has derived from case reports. Given that such infection is not routine, it can be inferred to be a kind of nosocomial and sporadic infection. Infection with *Tsukamurella* spp. is rare and mostly caused by contact with infected catheters [16]. Some reports regarding *Tsukamurella* infections have identified the sources of infections as other mycolic acid–containing actinomycetes. Lung disease in immunodeficient patients has long been attributed to *Tsukamurella* infection. The most commonly reported sources of *Tsukamurella* infection include bacteraemia [17], [18], [19], [20], [21], [22], [23], [24], meningitis [25], peritonitis [26], keratitis [27], [28], [29], cutaneous infection [30], conjunctivitis [23], [29], [31], brain abscess [32], respiratory tract infection [29], [33], [34], [35], [36], [37], [38], catheter-related bloodstream infection [16], [19], [23], [29], [39], [40], [41], [42], [43], [44] and acute otitis media [16]. In addition, *Tsukamurella* is a threat to people with immunodeficiency, including HIV infection [34], [45]. The risk factors for such infection are renal failure, foreign bodies (e.g. clinical equipment) and most importantly immunosuppressive diseases [46]. Tuberculosis-like syndromes and symptoms include acute bronchitis, bacteraemic pneumonia, productive cough, haemoptysis and weight loss [47]. The isolation history of *Tsukamurella* spp. is shown in Table 2.

**Table 2:** Isolation history of *Tsukamurella* spp

Species	Introduced in year	First isolation in:											G + C content (mol%)	Study
		Clinical specimens						Environmental samples						
		Mycetoma	Conjunctival swab	Lung	Sputum	Blood	Catheter	Soil	Deep-water marine sponge	Sludge foam	Insect	Venomous snake		
*T. tyrosinosolvens*^a^	1997					+							73.6+ 0.03%	Yassin, Rainey *et al.*, 1997
*T. hongkongensis*	2016		+			+							71.2 ± 2.3%	Teng, Tang *et al.,* 2016
*T. inchonensis*	1995			+		+							72%	Yassin, Rainey *et al.,* 1995
*T. pseudospumae*^a^	2004									+			ND	Nam, Kim *et al.,* 2004
*T. paurometabola*	1988	+									+		ND	Collins *et al.,* 1988
*T. pulmonis*^a^	1996				+								%69.8	Yassin, Rainey *et al.,* 1996
*T. sinensis*	2016		+			+							70.9 ± 2.2%	Teng, Tang *et al.,* 2016
*T. soli*	2010							+					70%	Weon, Yoo *et al.,* 2010
*T. spumae*	2003									+			72%	Nam, Chun *et al.,* 2003
*T. strandjordae*	2002					+							ND	Kattar, Cookson *et al.,* 2001
*T. serpentis*	2016											+	69.2 ± 1.5	Tang, Teng 2016

ND, not determined.

a See current taxonomy of *Tsukamurella* species.

## Identification Methods

### Phenotypic methods

Members of aerobic actinomycetes are increasing as a result of the discovery of new species, and clinical microbiologists will face problems identifying them. Phenotypic tests are the first way to distinguish among aerobic actinomycetes, although the spectra of these tests do not have all the varieties in laboratories to permit identification among species. For instance, analyzing whole-cell sugar is rarely used for identification in clinical laboratories. Staining of these bacteria in a medical laboratory is by Gram stain, which can differentiate a partially acid-fast organism from an acid-fast one. Most conventional and standard media are suitable for growing this bacterium. Blood agar, chocolate agar, brain–heart infusion agar, Sabouraud dextrose agar, Columbia agar with 5% defibrinated sheep's blood agar [12] and Löwenstein-Jensen medium will all support the growth of most aerobic actinomycetes. Aerobic conditions are recommended to better grow aerobic actinomycetes. *Tsukamurella* treatment by partially acid-fast stain shows weakly positive colony morphology; bacteria on conventional media are small and dry, with convex elevation, and form white, cream-coloured to orange colonies [48]. According to *Bergey's Manual of Systematic Bacteriology,* colonies of *T. paurometabola* are smooth and creamy and have a fried-egg appearance; other species can show a variety of colours. Optimal growth temperature is 25 to 37°C. As Steinhaus [5] has noted, semisolid medium with gelatin, carbohydrates and rabbit serum is the basic medium for isolation of *T. paurometabola* [15]. The species show partially acid-fast results, but some researchers believe that strongly acid-fast results have been observed in some species [3]. Matrix-assisted desorption ionization–time of flight mass spectrometry (MALDI-TOF MS) may also be performed to help with identification [49], [50].

### Molecular methods

**Fig. 1:**
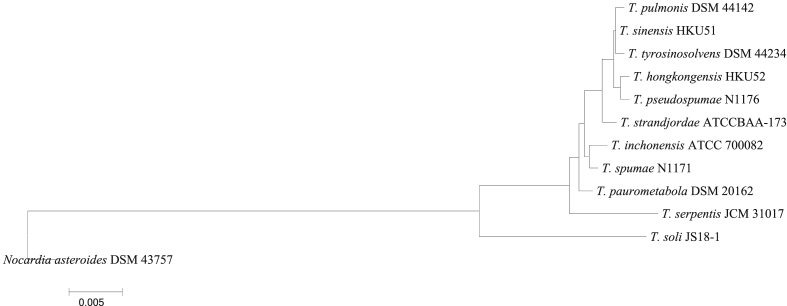
Full gene sequencing (∼1500 bp fragment) of 16S rRNA gene-based phylogenetic tree of *Tsukamurella* species (standard isolates) computed by neighbour-joining analyses and Kimura two-parameter model. Support of each branch as determined from 1000 bootstrap samples. Bar 0.005 indicates one nucleotide substitution per 100 nucleotides.

16S rRNA gene sequencing can be carried out to detect *Tsukamurella* spp. Park *et al.* [51] used primers 27F (5′-AGAGTTTGATCMTGGCTCAG-3′) and 1525R (5′-AAGGAGGTGWTCCARCC-3′) for the PCR amplification of the 16S rRNA gene. Through the PCR-mediated amplification of 16S rRNA gene and lipid analysis based on thin-layer chromatography, Yassin *et al.* [52] isolated *T. pulmonis.* Woo *et al.* [31] used the primers LPW57 (5′-AGTTTGATCCTGGCTCAG-3′) and LPW58 (5′-AGGCCCGGGAACGTATTCAC-3′). DNA-DNA hybridization is rarely used in clinical laboratories, although it is a standard molecular method for novel species identification. Via dot blot analysis, Kattar *et al.* [11] performed DNA-DNA hybridization and also carried out gas liquid chromatography, high-performance liquid chromatography and 16S rRNA gene sequencing with the primers 8FPL and DG74. Housekeeping genes such as *hsp65* (heat shock protein 65), *rpoB* (RNA polymerase, β subunit), *gyrB* (DNA gyrase, subunit B), *groEL* (molecular chaperone GroEL) and the 16S–23S internal transcribed spacer are used to identify aerobic actinomycetes [3]. Teng *et al.* [53] used 16S rRNA, *ssrA* (small stable RNA), *secA* (secretory), *rpoB* and *groEL* for differentiation of *Tsukamurella* species; results indicated that only 16S rRNA and *groEL* were effective in indicating the exact species. Teng *et al.* [50] recommended the use of PCR–restriction fragment length polymorphism (RFLP) and 16S rRNA gene sequencing analysis or other advanced molecular methods to differentiate *Tsukamurella* from other similar genus. Pérez *et al.* [54], [55] also used 16S rRNA analysis, with PA (5′-AGAGTTTGATCCTGGCTCAG-3′) and PLO6 R (5′-GCGCTCGTTGCGGGACTTA ACC-3′) and Tsuka1(5′-CTACCTGCGCGACAACATG-3′), as well as Tsuka2 (5′-CGATCGTCTTCTTGCGGATG-3′) as primers for *secA*1 genes on microorganisms in blood from bloodstream infections, which indicated *T. pulmonis.* According to the evidence of Teng *et al.* [8], [53], the 16S rRNA gene was unsuccessful in differentiating *T. sinensis* from some strains such as *T. pulmonis* and *T. tyrosinosolven;* furthermore, *secA*1 failed to differentiate between *Tsukamurella spumae* and *Tsukamurella pseudospumae.* Two primers used for the *hsp65* gene included TB11 (5′-ACCAACGATGGTGTGTCCAT-3′) and TB12 (5′-CTTGTCGAACCGCATACCCT-3′); the results indicated that *T. spumae* was the cause of an infection in that case report; for 16S rRNA gene sequencing, LPW27807 (5′-TGGCTCAGGACGAACGCT-3′) and LPW27808 (5′-GAGGTGATCCAGCCGCA-3′) were used [50]. PCR-RFLP was the first method adopted in *Tsukamurella* identification [50]. The 16S rRNA gene-based phylogenetic tree of *Tsukamurella* species was analysed by MEGA5 software [56] (Fig. 1).

## Antibiotic Susceptibility Testing and Treatment

In the literature, there is little information about sensitivity to antibiotics in the genus *Tsukamurella.* The best antibiotic susceptibility testing is microbroth dilution, which was introduced by the Clinical and Laboratory Standards Institute [57]*. Tsukamurella* spp. are resistant to penicillin, oxacillin, piperacillin/tazobactam and cephalosporins, which are prescribed for treatment of nontuberculous mycobacteria infection, whereas *Tsukamurella* is susceptible to amikacin, ciprofloxacin, imipenem, doxycycline, linezolid and sulfamethoxazole [47], [58]. Because of the insufficiency of guidelines regarding the treatment of *Tsukamurella* infection, the combination of β-lactam and aminoglycoside antibiotic agents and the removal of catheters has been recommended for improved outcomes. Therefore, as a related taxon, *Tsukamurella* can be inactivated by the ribosylation of the 23-OH group of antibiotics [59]. Effective results resulted from a combination of β-lactam or macrolide with aminoglycoside antibiotic agents for a long treatment period [47]. Susceptibility testing is necessary for proper treatment of *Tsukamurella* infections [16].

## Current Taxonomy of *Tsukamurella* Species

According to some new work by Teng *et al.* [50], some species such as *T. tyrosinosolvens, T. pseudospumae* and *T. pulmonis* have been misclassified. Too much similarity between some species of *Tsukamurella* in many molecular and phenotypic experiments was the reason that Teng *et al.* asked researchers to confirm the hypothesis that these three species in fact are the same type and misclassified. MALDI-TOF MS analysis, 16S rRNA gene sequencing, phylogenetic analysis, whole genome comparison, DNA-DNA hybridization and phenotypic characteristics are some of the studies Teng *et al.* performed. Research revealed that even though high similarity exists between *T. tyrosinosolvens* and *T. carboxydivorans* in the genomic analysis, some genomic islands and regions are present in the *T. tyrosinosolvens* genome. Further investigation and analysis indicated that these islands are mobile element proteins and other proteins related to phages. Although the *T. tyrosinosolvens* that they used their in research had been isolated from a patient with a cardiac pacemaker implant and the *T. carboxydivorans* had an environmental source (soil), more research is needed to confirm the pathogenic power of these genomic islands. After assessing similarity by G+C content, 16S rRNA gene sequencing, MALDI-TOF MS and phylogenomic analyses, reclassification was suggested for *T. spongiae* as *T. pulmonis, T. carboxydivorans* as *T. tyrosinosolvens* and finally *T. sunchonensis* as *T. pseudospumae* [50].

## Conclusion

To our knowledge, this is the first review of the literature of *Tsukamurella* species. Despite the lack of information on this genus and its status as a kind of saprophyte, it has been confirmed as a cause of opportunistic infections. Members of this genus are increasingly being identified, thus highlighting the need to clarify its features for improved cooperation between doctors and microbiologists. The pathogenic mechanism and antibiotic resistance of *Tsukamurella* are unknown and therefore require more research that involves clinical samples and new detection methods. It is hoped that in the near future new molecular methods can reveal different aspects of *Tsukamurella* for the promotion of clinical perspectives and development of enhanced treatment options.

## Conflict of Interest

None declared.
